# Long-Term Natural Outcomes of Simple Hemorrhage Associated with Lacquer Crack in High Myopia: A Risk Factor for Myopic CNV?

**DOI:** 10.1155/2018/3150923

**Published:** 2018-01-24

**Authors:** Bing Liu, Xiongze Zhang, Lan Mi, Ling Chen, Feng Wen

**Affiliations:** State Key Laboratory of Ophthalmology, Zhongshan Ophthalmic Center, Sun Yat-sen University, Guangzhou 510060, China

## Abstract

**Purpose:**

To investigate the relationship between simple hemorrhage (SH) associated with lacquer crack (LC) and myopic choroidal neovascularization (CNV) in high myopia.

**Methods:**

A cross-sectional evaluation including best-corrected visual acuity (BCVA), axial length, refractive error, color fundus photography, and spectral domain optical coherence tomography (SD-OCT) was performed in patients diagnosed with high myopia and SH. Fundus fluorescein angiography and indocyanine green angiography were performed if the eye was suspected with CNV.

**Results:**

Thirty-three eyes of 27 patients with SH were enrolled in the study. None of the eyes developed CNV at final examination following the occurrence of hemorrhage. Recurrent hemorrhage was observed in 36.5% of the eyes. Compared with the initial BCVA, the final BCVA was significantly improved (*P* < 0.001) and correlated with the integrity of the ellipsoid zone in SD-OCT. There was no significant difference in the final BCVA between group 1 (LC crossed the central fovea) and group 2 (no LC crossed the central fovea) (*P* = 0.299).

**Conclusions:**

SH associated with LC is not a risk factor for the development of myopic CNV in patients with high myopia. LCs have little influence on the final BCVA unless the integrity of the ellipsoid zone in the central fovea is disrupted.

## 1. Introduction

High myopia is a major cause of visual impairment and legal blindness worldwide, especially in Asian countries [1–3]. Among the various myopic fundus lesions, macular hemorrhage is a common vision-threatening complication in high myopia [4, 5]. Based on the presence of choroidal neovascularization (CNV), macular hemorrhage can be categorized into two types, hemorrhage secondary to myopic CNV and simple hemorrhage (SH).

There may be a relationship between SH and myopic CNV because the formation of both lesions is related to lacquer cracks (LCs) [6–8]. The occurrence of SH is generally associated with the formation of new LCs or progression of primary LCs [5, 6, 9, 10]. Most myopic CNVs seem to emanate from LCs or at least the areas adjacent to LCs, and the extension of LCs accompanies newly developed myopic CNV [11–14]. Therefore, some retina specialists have hypothesized that SH may be a risk factor for the development of myopic CNV. Several previous studies have reported the prognosis of SH in highly myopic eyes but have not investigated the relationship between SH associated with LC and myopic CNV; in addition, the results of these studies are limited by their short follow-up times [6, 15–17].

Thus, this study aims to investigate whether SH associated with LC is related to myopic CNV in highly myopic eyes. The long-term visual outcomes of highly myopic eyes with SH in a natural history are also evaluated in this study.

## 2. Materials and Methods

We performed a cross-sectional study. Patients with high myopia and SH who were referred to the Zhongshan Ophthalmic Center in Guangzhou, China, between January 1, 2010, and May 31, 2014, were enrolled. The study protocol was approved by the institutional review board of Zhongshan Ophthalmic Center of Sun Yat-sen University. The study was conducted in accordance with the tenets of the Declaration of Helsinki. Written informed consent was obtained from all patients in this study prior to enrollment.

Pathologic myopia was defined as a refractive error < −6 diopters (D) or an axial length > 26.0 mm. The diagnosis of SH was confirmed by ophthalmoscopic examination and fundus fluorescein angiography (FFA). The exclusion criteria included a history of other ocular disorders, such as dense cataract, glaucoma, diabetic retinopathy or other retinal vascular diseases, and age-related macular degeneration (AMD), and a history of vitreoretinal surgery, which might affect visual acuity. Because this study aimed to determine the natural course of SH in highly myopic eyes, patients who received any treatments for myopic fundus lesions following SH were also excluded.

Patient demographics, initial best-corrected visual acuity (BCVA), axial length, refractive error, color fundus photography, FFA imaging, and a history of follow-up were recorded from patient medical records.

A cross-sectional evaluation of all patients was performed and included BCVA using a Snellen chart, axial length using IOL Master (Carl Zeiss Meditec, Oberkochen, Germany), refractive error, slit lamp examination, dilated fundus examination by indirect ophthalmoscopy (+90 D), color fundus photography, and spectral domain optical coherence tomography (SD-OCT; Spectralis HRA + OCT, Heidelberg Engineering, Heidelberg, Germany) between June 1, 2017, and June 30, 2017. If the diagnosis of CNV could not be confirmed by SD-OCT (hyperreflective area above the damaged retinal pigment epithelial level with intraretinal fluid/increased foveal thickness and/or serous foveal detachment), FFA and indocyanine green angiography (ICGA) (Spectralis HRA + OCT, Heidelberg Engineering, Heidelberg, Germany) were performed. SD-OCT scanning and analysis of all study eyes were performed by one experienced investigator. Horizontal and vertical SD-OCT scans of 6 or 3 mm were centered on the fovea and the location of SH. The integrity of the ellipsoid zone within the 1 mm centered fovea was evaluated as continuous or discontinuous. In addition, the eyes were divided into group 1 (LC passed the central fovea) and group 2 (no LC passed the central fovea). Differences in visual acuity between the two groups were investigated.

### 2.1. Statistical Analysis

Data were processed and analyzed using SPSS 16.0 software (Inc., Chicago, IL, USA). For the analysis, Snellen BCVA data were transformed into equivalent logarithms of the minimum angle of resolution (logMAR) values. To evaluate the differences in visual acuity between group 1 and group 2, we performed independent *t*-tests for continuous variables and chi-square tests for categorical variables. Changes in visual acuity at each follow-up were analyzed using paired *t*-tests. A *P* value < 0.05 was considered statistically significant.

## 3. Results

### 3.1. Patients

We screened 319 files, and 58 patients were eligible. Among these patients, 3 could not be contacted, and 28 refused to participate in this study because of good visual acuity. Thus, 33 eyes of 27 patients with SH were enrolled in the study. There were no differences in age, gender, or geographic location between those who participated and those who did not participate. The clinical characteristics of the 27 patients are summarized in Table 1. The study included 13 men and 14 women. The mean duration since the initial visit was 50.4 ± 16.3 months, with a range of 36 months to 87 months. For all patients, the clinical diagnosis was made within 1 month from the onset of their visual symptoms, such as blurred vision and a fixed shadow in the front of the eye with or without distorted vision.

### 3.2. Patient Clinical Data Collected from Medical Records

The size of hemorrhages ranged from 0.25 to 1.5 disc diameters (DDs), and all hemorrhages covered the central fovea. The mean hemorrhage duration was 3.0 ± 1.0 months, with a range of 1 month to 6 months.

LCs were identified by ophthalmoscopy and FFA in 17 eyes (51.5%) at the initial examination. After hemorrhage absorption, LCs were observed in 32 eyes (97.0%) at the location of the initial hemorrhage, and the mean BCVA improved to 0.38 ± 0.26 logMAR (0 to 1.0 logMAR) from the initial examination. The difference in BCVA between the two time points was significant (*P* < 0.001).

Recurrent hemorrhages were recorded in 12 eyes (36.4%) from the medical records. Of these eyes, 3 had 3 recurrent hemorrhages, 1 had 2 recurrent hemorrhages, and 8 had one recurrent hemorrhage. The recurrent hemorrhages almost completely resolved within 4 months.

### 3.3. Patient Clinical Data at Cross-Sectional Examination

The mean refractive error was −14.7 ± 4.6 D, and the mean axial length was 30.1 ± 1.4 mm at the final examination. There were significant differences in both the refractive error and axial length between the initial and final examinations (all *P* < 0.001).

None of the eyes developed CNV at the final examination following the occurrence of hemorrhage.

At the final examination, LCs were observed in 32 eyes (97.0%), and 12 (37.5%) LCs crossed the central fovea. No LC was detected in 1 eye from the initial to the final examination.

**Figure 1:**
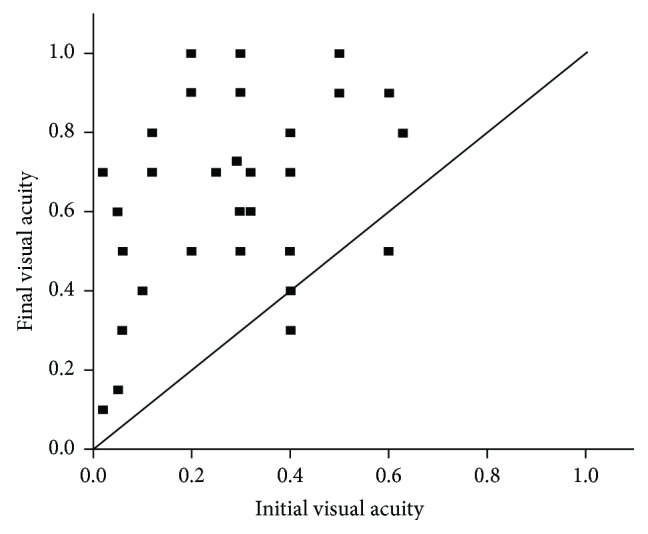
Initial and final Snellen BCVA of patients in this study. Dots on the line indicate unchanged visual acuity, dots above the line indicate improved visual acuity, and dots below the line indicate worse visual acuity.

**Figure 2:**
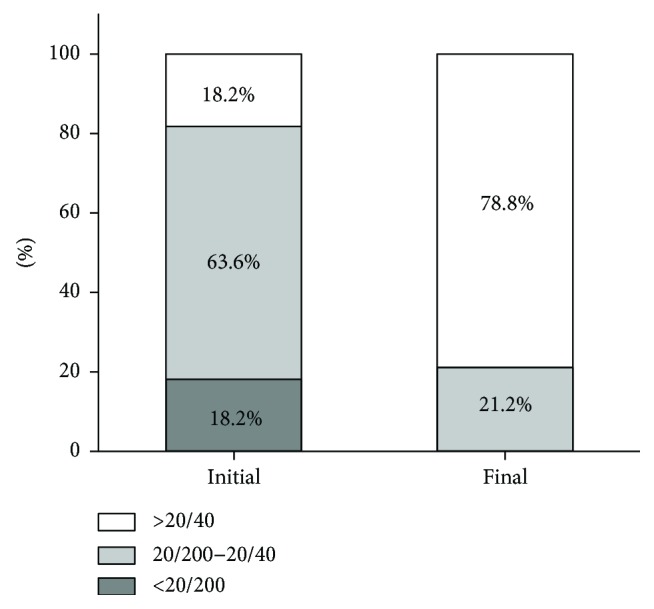
Distribution of the initial and final Snellen BCVA in this study.

Compared with the initial mean BCVA, the final mean BCVA was significantly improved (0.26 ± 0.21 logMAR; *P* < 0.001). The distributions of the initial and final Snellen VA are shown in Figures 1 and 2. The initial Snellen VA was 20/40 or greater in 6 eyes (18.2%), 20/200 to 20/40 in 21 eyes (63.6%), and 20/200 or less in 6 eyes (18.2%). The final Snellen VA was 20/40 or greater in 26 eyes (78.8%) and 20/200 to 20/40 in 7 eyes (21.2%). There was a significant difference in the number of eyes with a Snellen VA of 20/40 or greater between the initial and final visits (*P* < 0.001). Moreover, only two eyes had a worse VA at the final visit because of enlarged retinal pigment epithelium (RPE) and choroid atrophy.

### 3.4. Relationship between the Final BCVA and Lacquer Cracks

To investigate the relationship between the final BCVA and LCs, we divided 32 eyes with LCs into the following groups: group 1 (LC crossed the central fovea) and group 2 (no LC crossed the central fovea). Although the initial BCVA in group 1 was significantly greater than that in group 2, no significant difference was found in the final BCVA between the two groups (Table 2).

### 3.5. Relationship between the Final BCVA and OCT Findings

**Figure 3:**
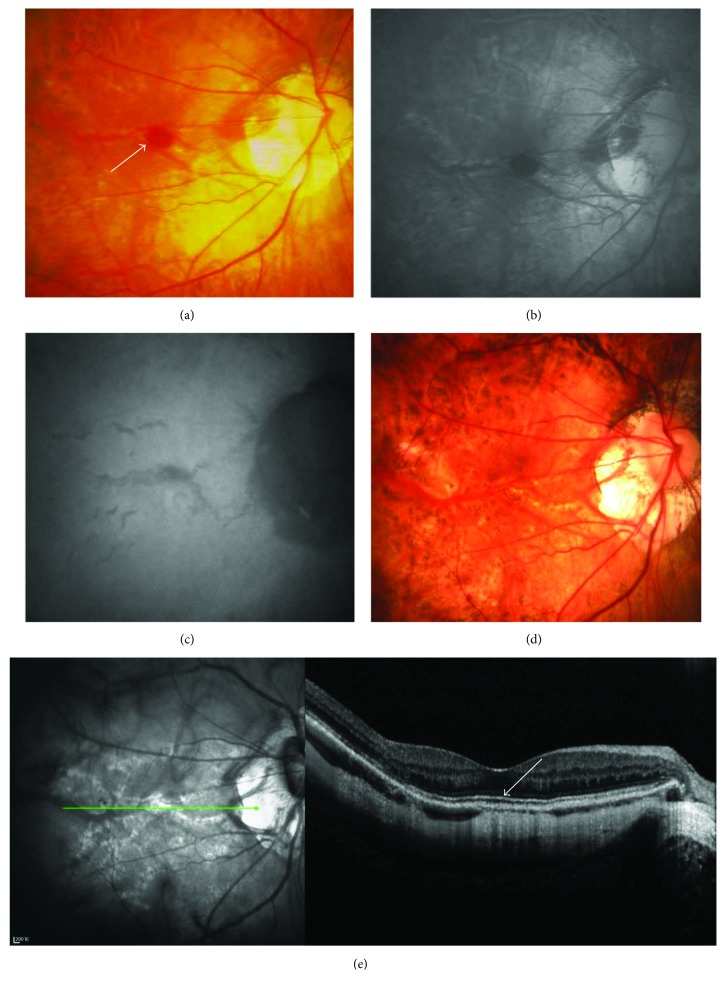
Multimodal imaging of an eye with a continuous ellipsoid zone. A 52-year-old woman was initially seen on September 2, 2011, with decreased visual acuity and a fixed shadow in her right eye. At the initial examination, the BCVA was 20/100, the refractive error was −8.75 D, and the axial length was 29.7 mm in her right eye. There was subretinal hemorrhage (white arrow) in the macular area of the right eye at the initial examination (a). The late phases of FFA and ICGA revealed a simple hemorrhage (b and c). During the follow-up period, recurrent hemorrhage and CNV were not detected. At the final examination (June 2, 2017), LCs were observed in the macular area by color fundus photography (d). An LC passing through the central fovea was easily observed with near-infrared reflectance imaging (e). Because the ellipsoid zone (white arrow) at the central fovea was continuous (e), the final BCVA of the eye was still good (20/25).

**Figure 4:**
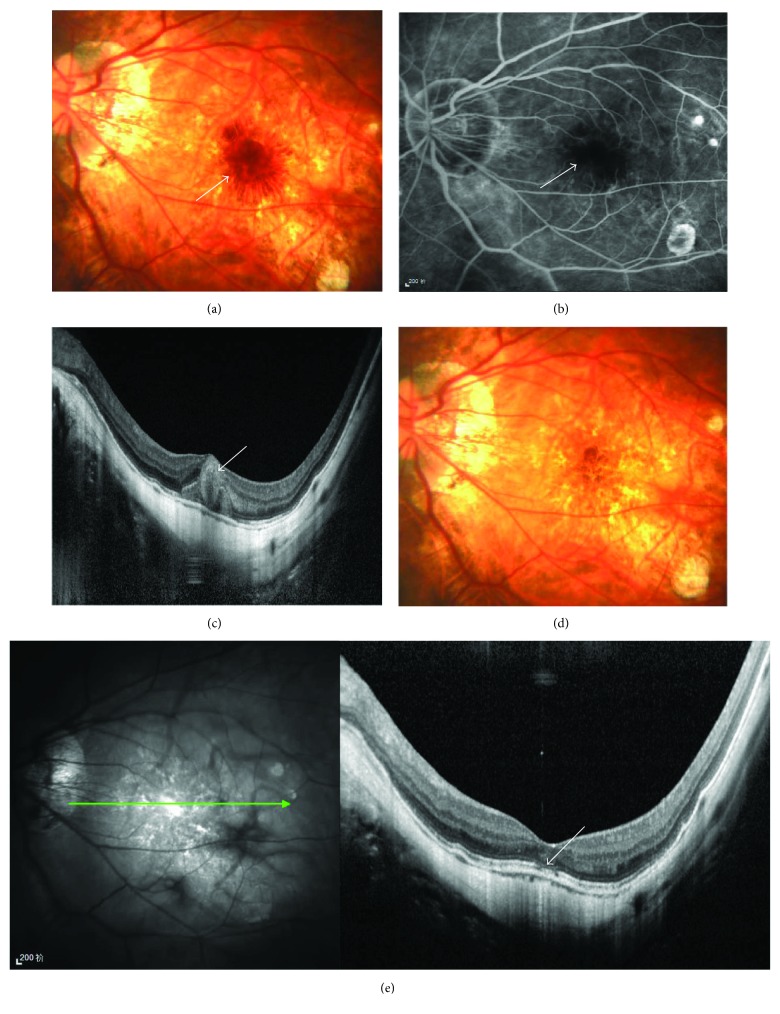
Multimodal imaging of an eye with a discontinuous ellipsoid zone. A 40-year-old woman was initially seen on December 3, 2013, with decreased visual acuity in her left eye. At the initial examination, the BCVA was 5/100, the refractive error was −16.5 D, and the axial length was 27.37 mm in her left eye. An obvious subretinal hemorrhage (white arrow) was observed in the macular area at the initial examination (a). The late phase of FFA indicated that it was a simple hemorrhage (white arrow) (b). SD-OCT showed that the hemorrhage (white arrow) was thick and reached beyond the ellipsoid zone (c). After 3 years of follow-up, no recurrent hemorrhage or CNV occurred. Because the ellipsoid zone (white arrow) at the central fovea was not continuous (e), the final BCVA of the eye was poor (20/100).

Of the 33 eyes, 27 had a continuous ellipsoid zone in the central fovea in OCT. Compared with eyes with a discontinuous ellipsoid zone, eyes with a continuous ellipsoid zone had a significantly improved final BCVA (0.48 ± 0.35 logMAR versus 0.21 ± 0.13 logMAR; *P* < 0.001, Figures 3 and 4).

## 4. Discussion

SH is a term used for macular hemorrhage without CNV that occurs in highly myopic eyes and is most likely caused by the mechanical rupture of Bruch's membrane and choriocapillaris complex [15, 16]. Although some researchers have suspected that SH may be a precursor of myopic CNV [15], the evidence is insufficient. A better understanding of the predictors of myopic CNV development may lead to more personalized treatment and help improve clinical outcomes and reduce recurrence [8, 18]. To investigate the relationship between SH associated with LC and myopic CNV, we performed a cross-sectional study.

In this study, no eyes with SH associated with LC developed CNV following hemorrhage occurrence. This result is consistent with the results of several previous studies [15, 16]. Goto et al. examined 20 eyes of 17 consecutive patients with SH and found that most of the patients had a good recovery, and no eye in the SH group developed CNV during the 1-year follow-up [15]. Moriyama et al. examined 31 eyes of 28 patients with high myopia and SH; they also found that no eye developed CNV during a mean follow-up of 17.7 months [16]. These results may be explained by differences in the pathogenesis between SH associated with LC and myopic CNV. LCs are caused by the mechanical rupture of Bruch's membrane. If both the choriocapillaris and Bruch's membrane rupture during formation or progression of LCs, then SH could be observed. The pathogenesis of myopic CNV remains controversial, and several theories, such as the mechanical theory, heredodegenerative theory, and hemodynamic changes in choroidal circulation [19–23], have been proposed. In our opinion, the development of myopic CNV may result from the interaction of multiple factors. LCs are necessary but not sufficient for the development of myopic CNV. The occurrence of myopic CNV may require other conditions mentioned above. Therefore, SH associated with LC is not a risk factor for the development of myopic CNV.

The visual prognosis of the myopic eyes with SH may be fair during a short follow-up period unless the hemorrhage recurs, atrophic scars develop, or retinochoroidal degeneration progresses [17]. In this study, similar results were observed in the long-term natural history. Most patients had good visual outcomes after at least 3 years of observation except for two eyes in which severe diffuse retinochoroidal atrophy was detected in the posterior fundus. In addition, recurrent hemorrhages were detected in 12 eyes (36.4%), indicating a high prevalence of recurrent hemorrhage in highly myopic eyes.

In this study, we also evaluated the relationship between final visual acuity and fundus changes. There was a significant correlation between the visual outcome and OCT findings. At the last visit, eyes with a continuous ellipsoid zone in the central fovea usually had better visual outcomes. This result is compatible with the findings of previous reports [15, 16, 24, 25]. For eyes with a discontinuous ellipsoid zone, the SH may be thick and reach beyond the ellipsoid zone; thus, severe damage to the retinal structure may limit visual recovery after hemorrhage absorption [16]. In addition, LCs had little effect on the final visual acuity when they crossed the central fovea. This finding is interesting and has not been previously reported. However, our OCT findings can explain this result. LCs are formed by ruptures in Bruch's membrane in which small hemorrhages may develop. After the hemorrhages were absorbed, there were usually no special findings in the layers of the RPE, Bruch's membrane, and choriocapillaris complex at the location of LCs in OCT. If the ellipsoid zone above the LCs was continuous, the eye with SH had a good visual outcome; otherwise, the visual acuity was poor. Thus, ellipsoid zone integrity but not LCs reflected the final outcomes of visual acuity. In addition, although 12 eyes had recurrent hemorrhages, their final visual acuity remained good. We found that these eyes had a continuous or relatively continuous ellipsoid zone in the central fovea in OCT. Thus, these findings also revealed the importance of the integrity of the ellipsoid zone in the central fovea for the final visual acuity.

Our study has several limitations that need to be considered. First, all patients were from a single institution; thus, a referral bias may exist. Second, a small number of patients participated in this study. However, to the best of our knowledge, this study is the first to evaluate the relationship between SH associated with LC and myopic CNV in high myopia in a long-term natural history.

In conclusion, in this study, we demonstrated that SH associated with LC is not a risk factor for the development of myopic CNV and that SH may recur in approximately 1/3 of eyes with high myopia. The long-term visual outcome of SH in high myopia was generally good, and it correlated with the integrity of the ellipsoid zone in OCT. In addition, LCs that passed through the central fovea had little influence on the final visual acuity unless the integrity of the ellipsoid zone in the central fovea was disrupted.

## Figures and Tables

**Table 1:** Patient clinical characteristics (*n* = 27).

Characteristics	
Age (years)	39.0 ± 8.5
Male/total (*n*, %)	13/27 (48.1)
Bilateral hemorrhage (*n*, %)	6/27 (22.2)
Refractive error (diopters)	
Initial	−13.2 ± 4.3
Final	−14.7 ± 4.6
Axial length (mm)	
Initial	28.7 ± 1.3
Final	30.1 ± 1.4
BCVA (logMAR)	
Initial	0.68 ± 0.40
Final	0.26 ± 0.21
Mean duration since initial visit (months)	50.4 ± 16.3
Mean duration of hemorrhage (months)	3.0 ± 1.0

**Table 2:** Comparison of clinical characteristics between group 1 and group 2.

	Group 1	Group 2	*P*
Eyes	12	20	—
Age (year)	38.7 ± 8.1	38.1 ± 8.1	0.968
Initial refractive error (diopters)	−13.0 ± 4.6	−13.4 ± 4.3	0.830
Initial axial length (mm)	28.3 ± 1.1	28.8 ± 1.5	0.339
Initial BCVA (logMAR)	0.76 ± 0.45	0.58 ± 0.26	0.017
Final BCVA (logMAR)	0.30 ± 0.24	0.18 ± 0.14	0.299
Eyes with recurrent hemorrhage (*n*, %)	5 (41.7%)	7 (35.0%)	0.800
